# Failure to Return for Posttest Counseling and HIV Test Results at the Prevention and Voluntary Testing and Counseling Centers of Douala, Cameroon: An Evaluation of a Routine Five-Year Program

**DOI:** 10.1155/2016/9720148

**Published:** 2016-01-26

**Authors:** Patrice Ngangue, Emmanuelle Bedard, Gerard Ngueta, Dieudonné Adiogo, Marie-Pierre Gagnon

**Affiliations:** ^1^Centre de Recherche du CHUQ, Axe Santé des Populations et Pratiques Optimales en Santé, 10 Rue de l'Espinay, QC, Canada G1L 3L5; ^2^Faculté des Sciences Infirmières, Université Laval, QC, Canada; ^3^Faculté des Sciences Infirmières, Université du Québec à Rimouski (UQAR), Campus de Levis, 1595 Boulevard Alphonse Desjardins, Levis, QC, Canada G6V 0A6; ^4^Département de Santé Environnementale et Santé au Travail, Institut de Recherche en Santé Publique de l'Université de Montréal (IRSPUM), Université de Montréal, Montréal, QC, Canada H3T 1A8; ^5^Faculté de Médecine et Sciences Pharmaceutiques, Université de Douala, BP 15253 Douala, Douala, Cameroon

## Abstract

This study examined the magnitude and time trends in failure to return (FTR) rates and the relation between FTR and individual characteristics, tests procedures, waiting period for the results, and HIV test results among people who were screened for HIV in the prevention and voluntary testing and counseling centers (PVTCCs) of six district hospitals of the city of Douala in Cameroon, between January 2009 and December 2013. It was a retrospective analysis of medical records. Among the 32,020 analyzed records, the failure to return (FTR) rate was 14.3%. Overall, people aged 50 years and over, those tested between 2011 and 2012, and those tested in the PVTCC of Bonassama were less likely to return for their results. Significant factors associated with FTR included being a housewife, having a positive/undetermined/requiring confirmation result, and provider-initiated testing and counseling (PITC). There was an increasing trend for FTR in the PVTCCs of Bonassama, New-Bell, Nylon, and Cité des Palmiers. HIV testing and counseling services in Douala district hospitals must be reorganized such that individuals tested for HIV receive their results on the same day of the test. Also counselors need to better alert clients concerning the importance of returning for their test results.

## 1. Introduction

In Sub-Saharan Africa, HIV testing and counseling (HTC) has been introduced in most health facilities, and it is recognized that HIV testing and especially return to receive results rates are very low [1–3]. HTC offers the opportunity for HIV-negative people as well as their partners to benefit from counseling in order to modify their risky sexual behaviors [4, 5]. HTC is also seen as the gateway to various other support and counseling services for HIV/AIDS, comprising other forms of treatment and psychosocial support [6]. Numerous studies conducted in Sub-Saharan Africa have found that the fear of a positive HIV result, stigmatization and discrimination, low educational level, low individual perception of risk, lack of knowledge about HIV/AIDS, low income, and young age were the main factors often associated with failure to return (FTR) for HIV test results. In these studies, the proportion of individuals who did not return for their result ranged from 10% to 50% depending on the country [7–9].

Located in the Gulf of Guinea, Cameroon is a Central Africa country with an estimated population of 20 million people [10]. This population is unevenly distributed within the national territory (48.8% in urban areas), with a greater concentration in the cities of Yaoundé (political capital) and Douala (economic capital). Cameroon's population is characterized by its extreme youth. Individuals under the age of 15 years and those between 15 and 49 years of age represent 44% and 47% of the population, respectively. Women represent 51% of the population [10]. Data from the 2011 Demographic and Health Survey (DHS 2011) show that 4.3% of people between 15 and 49 years of age are HIV-positive. HIV prevalence among women aged 15–49 (5.6%) is almost twice as high as for males of the same age bracket (2.9%) [11].

The government's response to HIV infection includes several strategies such as strengthening the prevention of transmission of HIV and STIs and access to care and treatment. One of the most important responses is HTC. Knowledge of HIV status is a priority. The highest number of people tested was reached in 2008 (40.34%). After a decline in screening in 2009 (24.64%), there has been an upward trend in the number of people tested since 2011, but this remains insufficient. Although the 2011–2013 operational plan aimed for the screening of 1,236,803 people in 2012, only 512,087 (41%) received counseling and underwent voluntary testing [12]. In addition, it is estimated that a significant proportion of infected women (30%) and infected men (36%) had never tested for HIV or had undergone testing but ignored the results [11].

Our study focused on the city of Douala, the capital of Cameroon's Littoral Region and the country's economic capital. The city has six district hospitals and is the most populated urban centre in Cameroon [10], with 1,907,479 habitants. According to the 2011 Cameroon Demographic and Health Survey (DHS 2011), the prevalence of HIV/AIDS reached 4.6% for people aged 15 to 49 years in the city of Douala. Women were particularly affected, with a prevalence of 6.4% as opposed to 2.6% for men [11]. As it is the case for the Sub-Saharan context, there is a lack of data on the proportion of people who do not return for their test results as well as the factors associated with FTR in the general population. Furthermore, there is no literature specifically addressing this issue in Cameroon. These data are crucial for purposes of informing health stakeholders and designing interventions to address this issue.

This study examines the magnitude and time trends in FTR rates and the relation between FTR and individual characteristics, tests procedures, waiting period for the results, and HIV test results among people who were screened for HIV in six district hospitals of Douala, Cameroon, between January 2009 and December 2013.

## 2. Methods

### 2.1. Setting and Study Population

The study population was composed of individuals aged 15 and over who had undergone both provider-initiated counseling and testing (PITC) and a voluntary confidential HIV test (VCT) between January 2009 and December 2013 in prevention and voluntary testing and counseling centers (PVTCCs) located in six district hospitals of Douala (Bonassama, Deido, New-Bell, Nylon, Cité des Palmiers, and Logbaba). These are integral components of district management units (DMU). These units were created within district hospitals to provide a link between the screening, management, and care for people living with HIV. Each unit is directed by an experienced nurse but is under a physician's supervision. The team also comprises at least two trained counselors.

### 2.2. HIV Testing and Counseling Procedure

At the PVTCC, the general strategy for HIV counseling and testing follows the guidelines suggested by the National AIDS Control Committee (NACC). According to these guidelines, individuals undergoing HIV testing are prepared and interviewed by a trained counselor during the pretest counseling. The aim of this discussion is to prepare the client for screening by explaining the meaning of an HIV test, evaluating risk behaviors, and addressing concerns (misinformation and/or misconception) about HIV infection. After informed verbal consent is obtained, the client is sent to the hospital laboratory where a blood sample is collected for rapid HIV antibody testing. Before the blood collection, the client has to pay for the test. The strategy consisted of a serial rapid testing algorithm. Nonreactive specimens at the first test were considered as true negatives. Reactive samples were evaluated with a second test (confirmatory test) and, if reactive, were considered as HIV antibody positive. Discordant specimens (undetermined) were resolved using a third different screening test (e.g., ELISA). After the HIV test, the client was instructed to return to the PVTCC for posttest counseling and to receive the result. The delay between collection of blood samples and availability of test results varied between the PVTCCs. The appointment was within 24 hours at 5 PVTCCs and within 48 hours in one such center (Bonassama). In the Bonassama PVTCC, after laboratory testing, the results must be validated by a biologist prior to being addressed by the counselor. In all six PVTCCs, the confirmatory test was not carried out on the same day as the first test. It required a second sample after pretest counseling and entailed additional charges.

### 2.3. Data Collection

We extracted available data from the PVTCC medical records, covering the period from January 2009 to December 2013. Extracted data were recorded in a standardized form. Independent variables included age, sex, marital status, occupation, PVTCC, year of testing, reason for testing (VCT or PITC), and test result. The outcome of interest, FTR, was determined when clients did not return for their results and if the nonreturn status was not recorded in the screening registry. There was one data record for each testing episode. Data from Cité des Palmiers and Logbaba District Hospitals were available for the years 2009 to 2011 only. In addition, we were not able to obtain data concerning the return for HIV test results from the Bonassama District Hospital for the years 2012 and 2013.

### 2.4. Statistical Analysis

All analyses were performed using SAS software (version 9.3 SAS Institute Inc., Cary, NC). Baseline characteristics of clients across the PVTCCs were described using Pearson's chi-squared test. The ANOVA procedure with post hoc Bonferroni test was used to compare the mean age of participants across PVTCCs. Pearson's chi-squared test was also used to estimate the prevalence of FTR and to evaluate the bivariate association between FTR and each independent variable. To assess the trend in the likelihood for FTR, year of test was used as a nominal variable and then introduced into the logistic regression model. The backward stepwise logistic procedure was used to identify the predictors of FTR. The odds ratios and their 95% confidence interval (CI) were then computed. For all analyses, a *p* value of 0.10 was used to identify significant determinants of FTR. Subsequently, we estimated the predicted probability of failure to return, and this was plotted by year to graphically assess the presence of a trend from 2009 to 2013.

In this study, we were not able to obtain any data concerning the return for results in the PVTCC of Bonassama District Hospital for 2012 and 2013. Due to the fact that these missing values mainly affected the outcome of interest, no specific treatment was applied in order to deal with this issue. However, given that clients of Bonassama District Hospital differed from the clients of other centers in most independent variables, we conducted a sensitivity analysis by excluding subjects from this center (see supplementary file in Supplementary Material available online at http://dx.doi.org/10.1155/2016/9720148).

This study was approved by the National Ethics Committee of Cameroon and the Human Research Ethics Committee of Laval University, Québec, Canada.

## 3. Results

During the study period, we extracted information from 32,022 medical records of clients aged 15 and above. Overall, 4,126 clients failed to return for their test results, leading to a FTR rate of 14.3%.

Table 1 summarizes the characteristics of the study population by PVTCC.

Although all PVTCCs are located in the city of Douala, we did not find similarities in the characteristics of clients except as concerns marital status. The median age ranged from 29 to 32 years of age. Women were more likely to be tested in the PVTCCs of Deido (17.2%), New-Bell (28.8%), Cité des Palmiers (12.6%), and Logbaba (2.1%). Across the PVTCCs, the majority of clients tested were aged 20 to 29 years and self-employed. Most clients were tested voluntarily.

Table 2 presents the distribution of the prevalence of FTR for HIV test results by client characteristics. The FTR rate did not differ significantly by gender or by reason for testing. It was 14.3% for women and men, as well as VCT and PITC clients. The FTR rate increases significantly with age (*p* = 0.0015). For example, clients aged 20 to 29 years were more likely to return for their results (13.3% FTR) than those aged 50 to 59 years (16.2% FTR). Concerning marital status, divorcees (20.0%) were least likely to return for their results than other groups (*p* < 0.0001). Moreover, the FTR rate was 15.8% among HIV-positive clients, 21.7% among clients with undetermined test results, and 57.9% among clients with results which had to be confirmed.

Table 3 presents factors associated with FTR. Multivariate analysis showed that age, employment, PVTCC, reason for testing, and year of testing were the predictors of FTR. Clients aged 20–29, 30–39, and 40–49 years were more likely to return for their results compared to those aged 50 years and over. Concerning employment, only retirees were more likely to return for their results compared to other categories of employment. These results are statistically significant for clients aged 20–29 years (aOR 0.82; 95% CI: 0.69–0.98) and housewives (aOR 1.24; 95% CI: 1.09–1.42). Clients with positive HIV test results (aOR = 1.31; 95% CI: 1.19–1.42), with undetermined HIV test results (aOR = 1.99; 95% CI: 1.50–2.65), and with a result which had to be confirmed (aOR = 10.7; 95% CI: 8.55–13.45) were more likely not to return than those who tested HIV-negative. VCT clients were also more likely to return for their results than provider-initiated counseling and testing clients (aOR = 0.8; 95% CI: 0.75–0.91).

Table 4 presents the time trends of FTR by year and PVTCC, related to age, sex, marital status, occupation, and reason for testing of clients tested. The FTR trend varies by year depending on the PVTCC. For example, for the Bonassama center, the likelihood for FTR increased dramatically between 2009, 2010 (aOR 1.82; 95% CI: 1.40–2.35), and 2011 (aOR. 16.94; 95% CI: 11.92–24.08). For the PVTCC of New-Bell, the likelihood for FTR decreased between 2009 and 2010 but increased gradually from 2011 (aOR 1.64, 95% CI: 1.18–2.28) until 2013 (aOR 2.37; 95% CI: 1.86–3.02). For the PVTCC of Nylon, the likelihood for FTR rate decreased from 2009 to 2012 but increased in 2013. There was an increasing likelihood for FTR for the PVTCC of Cité des Palmiers in 2013. In contrast, the likelihood for FTR decreased for the PVTCC of Deido and Logbaba. As a whole, missing values in FTR represent 9.7% of observations. They were due only to the Bonassama PVTCC which did not report FTR data in 2012 and 2013 (see supplement 1). Sensitivity analysis conducted by excluding subjects of the Bonassama centre did not show any changes in factors statistically associated with FTR. Gender and marital status become predictors of FTR, whereas employment was no longer a predictor. However, none of these factors proved to be statistically significant.

## 4. Discussion

In our study, among the 32,022 people who were tested for HIV in the six district hospitals of Douala between 2009 and 2013, 14.3% did not return for their results. Although quite high, the FTR found in our study is lower than the rate found by Sesay and Chien (30.1%) in a study conducted in Gambia in 2012 [9]. Interestingly, this rate was much higher than that of Laanani et al., in a study conducted in France in 2014 where FTR was only 6.5% [13]. However it is important to note that, in our study, the FTR rate varied according to the PVTCC, some clients' characteristics, the test result, and the year of testing.

Despite the availability of rapid tests and their ease of use, none of the PVTCCs delivers the results on test day. Results are available within 24 hours after the test in 5 PVTCCs and up to 48 hours after the test at the Bonassama PVTCC. Indeed, the rapid tests are not performed within the PVTCCs but rather in the district hospital's lab. Due to the workload and probability of lack of personnel, the blood samples are tested only later in the afternoon when the clients are gone. Moreover, at the Bonassama centre, a biologist must validate the results. The combination of these problems might explain why clients who were tested at the Bonassama PVTCC had significantly higher FTR rates compared to the other PVTCCs. Indeed, several studies have demonstrated that clients prefer to receive their result the same day as the test [1, 14]. The simple fact of having to go back to the testing center to receive results increases screening costs entailed by transportation and waste of time resulting from screening [2, 15]. In addition, the waiting period associated with fear of the result itself leads to anxiety, thus increasing the FTR [16].

In our study, age, employment, test result, and reason for the initiation of the test were factors significantly associated with FTR. Retirees and those under 50 years of age were more likely to return for their results. These study results differ from what is reported in the literature. According to several authors, young people often do not return for their test results, as they are less informed about HIV than adults [17–19]. Otherwise, individuals with stable jobs often have specific knowledge about HIV. These individuals have a good understanding of the importance of testing and are more likely to return for their results [7, 20].

Most clients were tested at the request of a healthcare provider, probably because of clinical suspicion. The association between positive test results and FTR had been found in previous studies, both in Africa [7] and in the USA [21]. In this regard, the literature suggests that people who believe themselves to be HIV infected develop stress and anxiety which in turn prevents the return for result and posttest counseling [7, 16, 21]. Although not yet demonstrated by other studies, we believe that this reason might be valid for people with an “undetermined” or to “be confirmed” result.

We observed an increasing trend of FTR by years for some PVTCCs (Bonassama, New-Bell, and Cité des Palmiers). This trend is reversed for other PVTCCs, for which a decline in FTR is observed (Deido and Logbaba). Organizational factors such as motivation of staff, the availability of antiretroviral therapy, and the relationship of trust between clients and counselors could explain these differences as mentioned in some studies [2, 7, 8].

Our results certainly show that actions were taken to improve VTC. However, these measures should be strengthened through evidence-based targeted strategies in order to effectively and sustainably circumvent this situation in Douala.

Our study had some limitations. We could not get any information on sexual behaviors of tested individuals, as this information was not documented in screening records. The possibility to consider these factors could have enriched our analysis. Indeed, the literature suggests that people with risky behaviors believe themselves to be HIV infected and develop fear or anxiety in regard to the test outcome. This psychological state prevents them from returning for their test results even if they had the courage to get tested [3, 18].

## 5. Conclusion

Our results made it possible to define the proportion of FTR and posttest counseling in the city of Douala and to identify associated factors. Although not generalizable to the entire population, these results showed that FTR represented a major problem in a country with a high HIV prevalence. This leads us to suggest rapid implementation of effective strategies, including better organization of screening services so that test results are available and provided to people during the same visit. This reorganization should consider the specificities and needs of each PVTCC. It must be done in association with all stakeholders. In addition, although it is difficult to establish a profile of people who may fail to return for their result, counselors should systematically assess patient's self-perceived risk and report risk behaviors during pretest counseling sessions. They should also be trained and equipped to identify and adapt their message for people likely to have “positive,” “undetermined,” or “requiring confirmation” status. The use of a risk assessment tool adapted to the Cameroon context and culture, such as the behavioral risk assessment tool (BRAT) in USA, could enable better targeting of groups at risk for FTR. Therefore, people would be encouraged to return for their results and benefit from posttest counseling.

## Supplementary Material

In the table, the results of the sensitivity analysis conducted by excluding data of the clients of the PVTCC of Bonassama, given their differences from the clients of others centers in most of independent variables.
In the forest plot, the predicted probability of failure to return by year to graphically assess the presence of trend from 2009 to 2013.

## Figures and Tables

**Table 1:** Baseline characteristics of clients testing through Douala PVTCC from January 1, 2009, to December 31, 2013 (column percentages do not include missing values).

	Bonassama	Deido	New-Bell	Nylon	Cité des Palmiers	Logbaba	*p* value
*N*	4,748	5,480	8,819	8,416	3,902	657	
Sex							<0.0001
Men	2,080 (16.0)	2,211 (17.0)	3,333 (25.7)	3,603 (27.7)	1,505 (11.6)	262 (2.0)	
Women	2,662 (14.0)	3,267 (17.2)	5,473 (28.8)	4,808 (25.3)	2,396 (12.6)	395 (2.1)	
Missing	6 (0.4)	2 (0.0)	13 (0.1)	5 (0.0)	0 (0.0)	0 (0.0)	
Median age (IQR)	31 (25–41)	30 (25–38)	30 (24–38)	32 (26–40)	29 (24–37)	31 (25–39)	<0.0001
Age groups (years)							<0.0001
<20	190 (13.2)	183 (12.7)	548 (38.2)	225 (15.7)	270 (18.8)	20 (1.4)	
20–29	1,841 (13.8)	2,405 (18.1)	3,740 (28.1)	3,224 (24.2)	1,830 (13.7)	276 (2.1)	
30–39	1,357 (14.3)	1,747 (18.5)	2,523 (26.6)	2,684 (28.3)	958 (10.1)	201 (2.1)	
40–49	753 (17.0)	649 (14.6)	1,079 (24.4)	1,410 (31.8)	440 (9.9)	98 (2.2)	
50–59	412 (18.6)	335 (15.1)	527 (23.8)	644 (29.1)	261 (11.8)	37 (1.7)	
≥60	173 (15.3)	160 (14.2)	401 (35.5)	228 (20.2)	142 (12.6)	25 (2.2)	
Missing	24 (0.4)	17 (0.3)	183 (2.0)	8 (0.0)	5 (0.0)	7 (1.0)	
Marital status							
Single	2,603 (14.1)	4,231 (22.9)	4,500 (24.4)	4,121 (22.4)	2,943 (16.0)	11 (0.1)	Not valid
Married	1,266 (14.9)	1,016 (11.9)	3,214 (37.7)	2,167 (25.4)	850 (9.9)	3 (0.0)	
Separated	27 (10.7)	21 (8.4)	74 (29.5)	126 (50.2)	3 (1.2)	0 (0.0)	
Living common law	425 (18.5)	32 (1.4)	595 (25.9)	1,241 (54.1)	0 (0.0)	0 (0.0)	
Widowed	304 (13.6)	152 (11.8)	259 (20.1)	472 (36.6)	100 (7.7)	2 (0.2)	
Missing	97 (2.0)	0 (0.0)	0 (0.0)	0 (0.0)	2 (0.0)	0 (0.0)	
Employment							<0.0001
Student	852 (14.9)	1,278 (22.5)	1,118 (19.7)	1,095 (19.3)	1,203 (21.2)	140 (2.5)	
Unemployed	182 (9.6)	246 (13.0)	694 (36.7)	692 (36.6)	67 (3.5)	11 (0.6)	
Employed	943 (18.2)	1,062 (20.5)	1,026 (19.8)	1,294 (25.0)	713 (13.8)	133 (2.6)	
Self-employed	1,798 (14.4)	1,827 (14.6)	3,551 (28.5)	3,909 (31.3)	1,153 (9.3)	233 (1.8)	
Housewife	823 (16.1)	853 (16.7)	1,615 (31.5)	1,130 (22.1)	589 (11.5)	114 (2.2)	
Retired	66 (20.4)	53 (16.4)	73 (22.6)	71 (21.9)	57 (17.7)	3 (0.9)	
Missing	81 (1.7)	0 (0.0)	0 (0.0)	0 (0.0)	0 (0.0)	0 (0.0)	
Reason for testing							<0.0001
Voluntary screening	3,207 (12.9)	4,375 (17.7)	7,359 (29.7)	6,697 (27.0)	2,485 (10.0)	645 (2.6)	
Clinical suspicion	1,119 (16.4)	1,105 (16.2)	1,460 (21.4)	1,719 (25.2)	1,412 (20.7)	12 (0.2)	
Missing	421 (8.8)	0 (0.0)	0 (0.0)	0 (0.0)	0 (0.0)	0 (0.0)	
Participants per year							
2009	416 (13.7)	769 (25.3)	1,084 (35.7)	767 (25.3)	0 (0.0)	0 (0.0)	
2010	793 (21.8)	433 (11.9)	825 (22.7)	1,580 (43.5)	0 (0.0)	0 (0.0)	
2011	420 (11.9)	963 (27.4)	596 (16.9)	1,542 (43.8)	0 (0.0)	0 (0.0)	
2012	1,861 (17.6)	1,512 (14,3)	3,268 (31.0)	2,212 (20.9)	1,420 (13.5)	286 (2.7)	
2013	1,258 (11.2)	1,803 (16.0)	3,046 (27.0)	2,315 (20.5)	2,482 (22.0)	371 (3.3)	

**Table 2:** Prevalence of failure to return (FTR) by client characteristics.

	*N* (%)	Prevalence of FTR	95% confidence interval	*p* for bivariate association
Sex				0.8821
Men	12,994 (40.6)	14.3	13.7, 14.9	
Women	19,001 (59.4)	14.3	13.8, 14.8	
Age groups (years)				0.0015
<20	1,436 (4.5)	14.4	12.6, 16.2	
20–29	13,316 (41.6)	13.3	12.7, 13.9	
30–39	9,470 (29.6)	14.7	14.0, 15.4	
40–49	4,429 (13.8)	15.1	14.0, 16.2	
50–59	2,216 (6.9)	16.2	14.6, 17.7	
≥60	1,129 (3.6)	15.4	13.3, 17.5	
Matrimonial status				0.0002
Single	18,409 (59.9)	13.7	13.2, 14.2	
Married	8,516 (27.7)	15.4	14.6, 16.2	
Separated	251 (0.8)	20.0	15.1, 24.9	
In couple	2,293 (7.4)	14.8	13.3, 16.3	
Widowed	1,289 (4.2)	16.5	14.5, 18.5	
Occupation				<0.0001
Student	5,686 (18.5)	12.3	11.4, 13.2	
Unemployed	1,892 (6.2)	15.4	13.8, 17.0	
Employed	5,171 (16.9)	13.4	12.5, 14.3	
Self-employed	12,471 (40.7)	14.7	14.1, 15.3	
Housewife	5,124 (16.7)	15.8	14.8, 16.8	
Retired	323 (1.0)	14.3	10.5, 18.1	
Center				<0.0001
Bonassama	4,748 (14.8)	48.6	47.2, 50.0	
Deido	5,480 (17.1)	10.8	9.9, 11.6	
New-Bell	8,819 (27.5)	15.1	14.4, 15.8	
Nylon	8,416 (26.3)	13.1	12.4, 13.8	
Cité des Palmiers	3,902 (12.2)	6.7	5.9, 7.5	
Logbaba	657 (2.1)	7.2	5.2, 9.2	
Reason for testing				0.8651
Voluntary screening	24,768 (78.4)	14.3	13.9, 14.7	
Clinical suspicion of HIV infection	6,827 (21.6)	14.3	13.5, 15.1	
Results of HIV test				<0.0001
Positive	6,765 (23.3)	15.8	14.9, 16.7	
Negative	21,578 (74.3)	12.9	12.5, 13.3	
To be confirmed	385 (1.3)	57.9	53.0, 62.8	
Undetermined	327 (1.1)	21.7	17.2, 26.2	

**Table 3:** Predictors of failure to return for HIV test results.

	*N* (%)	OR (95% CI)
Age groups (years)		
<20	1,436 (4.5)	1
20–29	13,316 (41.6)	0.82 (0.69, 0.98)
30–39	9,470 (29.6)	0.92 (0.76, 1.11)
40–49	4,429 (13.8)	0.89 (0.73, 1.09)
50–59	2,216 (6.9)	1.00 (0.80, 1.24)
≥60	1,129 (3.6)	1.11 (0.84, 1.47)
Occupation		
Student	5,686 (18.5)	1
Unemployed	1,892 (6.2)	1.11 (0.94, 1.31)
Employed	5,171 (16.9)	1.04 (0.90, 1.19)
Self-employed	12,471 (40.7)	1.11 (0.99, 1.24)
Housewife	5,124 (16.7)	1.24 (1.09, 1.42)
Retired	323 (1.0)	0.90 (0.60, 1.36)
Center		
Bonassama	4,748 (14.8)	1
Deido	5,480 (17.1)	0.12 (0.10, 0.13)
New-Bell	8,819 (27.5)	0.17 (0.15, 0.20)
Nylon	8,416 (26.3)	0.14 (0.13, 0.16)
Cité des Palmiers	3,902 (12.2)	0.07 (0.06, 0.09)
Logbaba	657 (2.1)	0.53 (0.13, 2.14)
Reason for testing		
Voluntary screening	24,768 (78.4)	1
Clinical suspicion of HIV infection	6,827 (21.6)	1.45 (1.31, 1.60)
Year of test		
2009	3036	1
2010	3631	1.02 (0.89, 1.17)
2011	3521	1.37 (1.20, 1.58)
2012	10559	1.16 (1.02, 1.33)
2013	11275	0.96 (0.84, 1.10)

**Table 4:** Adjusted association between year of test and the likelihood for failure to return, by PVTCC^†^.

	Bonassama	Deido	New-Bell	Nylon	Cité des Palmiers	Logbaba
Year of test						
2009	1	1	1	1		
2010	1.82 (1.40, 2.35)	1.09 (0.83, 1.42)	0.91 (0.64, 1.28)	0.98 (0.76, 1.26)		
2011	16.94 (11.92, 24.08)	0.12 (0.08, 0.17)	1.64 (1.18, 2.28)	0.97 (0.75, 1.25)		
2012	NA	0.38 (0.30, 0.48)	2.65 (2.09, 3.37)	0.92 (0.72, 1.17)	1	1
2013	NA	0.13 (0.10, 0.17)	2.37 (1.86, 3.02)	1.01 (0.79, 1.28)	1.23 (0.94, 1.61)	0.10 (0.04, 0.24)
*p* for trend	<0.0001	<0.0001	<0.0001	0.9971	NA	NA

^†^Adjusted for age, sex, marital status, occupation, and reason of test.
