# Supplementary material for: Failure to Return for Posttest Counseling and HIV Test Results at the Prevention and Voluntary Testing and Counseling Centers of Douala, Cameroon: An Evaluation of a Routine Five-Year Program
Source: AIDS Res Treat. 2016 Jan 26;2016:9720148. doi: 10.1155/2016/9720148 (PMC4746278; doi:10.1155/2016/9720148)

**Table**: Strong predictors for failure to return

|  | **N (%)** |  | **OR (95% CI)** |
| --- | --- | --- | --- |
| **Sex** |  |  |  |
| Women |  |  | 1 |
| Men |  |  | 1.09 (1.01, 1.18) |
| **Age groups (Years)** |  |  |  |
| < 20 |  |  | 1 |
| 20 - 29 |  |  | 0.85 (0.71, 1.02) |
| 30 - 39 |  |  | 0.97 (0.80, 1.17) |
| 40 - 49 |  |  | 0.92 (0.74, 1.13) |
| 50 - 59 |  |  | 1.09 (0.86, 1.37) |
| ≥ 60 |  |  | 1.10 (0.83, 1.37) |
| **Matrimonial status** |  |  |  |
| Single |  |  | 1 |
| Married |  |  | 1.05 (0.95, 1.15) |
| Separated |  |  | 1.42 (0.99, 2.03) |
| Living common law |  |  | 0.86 (0.74, 1.00) |
| Widowed |  |  | 0.96 (0.77, 1.18) |
| **Center** |  |  |  |
| Deido |  |  | 1 |
| New-Bell |  |  | 1.43 (1.29, 1.60) |
| Nylon |  |  | 1.27 (1.13, 1.42) |
| Cité des palmiers |  |  | 0.60 (0.51, 0.70) |
| Logbaba |  |  | 4.38 (1.09, 17.63) |
| **Reason for testing** |  |  |  |
| Voluntary screening |  |  | 1 |
| Clinical suspicion of HIV infection |  |  | 1.45 (1.31, 1.60) |
| **Year of test** |  |  |  |
| 2009 |  |  | 1 |
| 2010 |  |  | 0.90 (0.77, 1.06) |
| 2011 |  |  | 0.68 (0.58, 0.80) |
| 2012 |  |  | 0.88 (0.77, 1.00) |
| 2013 |  |  | 0.72 (0.63, 0.83) |

**Probability of failure to return, by year**


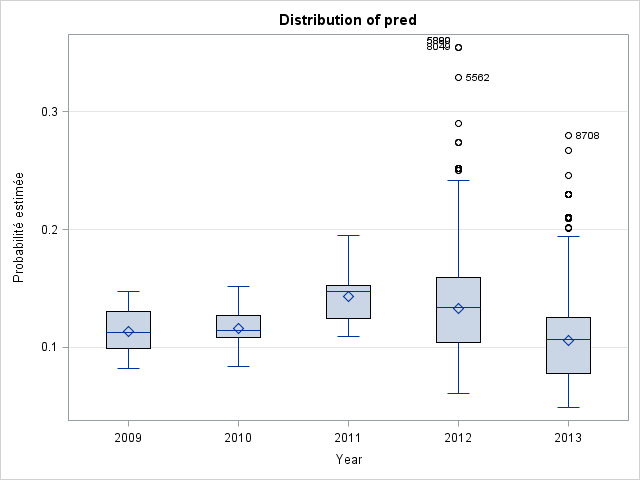

Supplement: Supplementary file 1 — In the table, the results of the sensitivity analysis conducted by excluding data of the clients of the PVTCC of Bonassama, given their differences from the clients of others centers in most of independent variables. In the forest plot, the predicted probability of failure to return by year to graphically assess the presence of trend from 2009 to 2013. [file 9720148.f1.doc]
